# Relationship between Milk Microbiota, Bacterial Load, Macronutrients, and Human Cells during Lactation

**DOI:** 10.3389/fmicb.2016.00492

**Published:** 2016-04-20

**Authors:** Alba Boix-Amorós, Maria C. Collado, Alex Mira

**Affiliations:** ^1^Department of Health and Genomics, Center for Advanced Research in Public Health, FISABIO FoundationValencia, Spain; ^2^Department of Biotechnology, Institute of Agrochemistry and Food Technology, Spanish National Research CouncilValencia, Spain

**Keywords:** human microbiome, breast milk, lactation, qPCR, flow cytometry, somatic cells, 16S rRNA, bacterial load

## Abstract

Human breast milk is considered the optimal nutrition for infants, providing essential nutrients and a broad range of bioactive compounds, as well as its own microbiota. However, the interaction among those components and the biological role of milk microorganisms is still uncovered. Thus, our aim was to identify the relationships between milk microbiota composition, bacterial load, macronutrients, and human cells during lactation. Bacterial load was estimated in milk samples from a total of 21 healthy mothers through lactation time by bacteria-specific qPCR targeted to the single-copy gene fusA. Milk microbiome composition and diversity was estimated by 16S-pyrosequencing and the structure of these bacteria in the fluid was studied by flow cytometry, qPCR, and microscopy. Fat, protein, lactose, and dry extract of milk as well as the number of somatic cells were also analyzed. We observed that milk bacterial communities were generally complex, and showed individual-specific profiles. Milk microbiota was dominated by *Staphylococcus, Pseudomonas, Streptococcus*, and *Acinetobacter. Staphylococcus aureus* was not detected in any of these samples from healthy mothers. There was high variability in composition and number of bacteria per milliliter among mothers and in some cases even within mothers at different time points. The median bacterial load was 10^6^ bacterial cells/ml through time, higher than those numbers reported by 16S gene PCR and culture methods. Furthermore, milk bacteria were present in a free-living, “planktonic” state, but also in equal proportion associated to human immune cells. There was no correlation between bacterial load and the amount of immune cells in milk, strengthening the idea that milk bacteria are not sensed as an infection by the immune system.

## Introduction

Human milk is a complex fluid adapted to satisfy the nutritional requirements of the infant, and also protective compounds which help to create the right microenvironment for gut development and maturation of the immune system (Petherick, 2010; Walker, 2010). More recently, milk has been recognized to host commensal and potential probiotic bacteria, which together with milk's growth factors and other components may have health implications. For example, they could be involved in the digestion of nutrients, facilitating the digestion process, although the most likely role for these microorganisms is immune modulation (Fernández et al., 2013) Culture-dependent methods have long confirmed the presence of viable bacteria in aseptically collected samples (Heikkilä and Saris, 2003). However, an important part of the species has not yet been cultured under laboratory conditions, and subsequently the diversity of human milk could be underestimated by classical approaches. Although partial contamination from skin microbes occurs, the presence of strictly anaerobic species such as *Bifidobacterium, Clostridium*, and some *Bacteroides* spp., which are absent in the skin microbiota, supports that breast milk hosts a unique microbiome (Hunt et al., 2011; Cabrera-Rubio et al., 2012a; Jost et al., 2013). Accumulating evidence suggeststhat milk microbiota is influenced by perinatal factors such as mode of delivery, lactation time, gestational age, maternal health, or geographical locations (Khodayar-Pardo et al., 2014; Cabrera-Rubio et al., 2016).

It has been estimated that an infant consumes ~800 mL/day, ingesting between 1 × 10^4^ and 1 × 10^6^ bacteria daily (Heikkilä and Saris, 2003) but those data were based on culture techniques and may have underestimated the total load of microorganisms. Other non culturable-dependent methods, such as molecular techniques or cytometry should be implemented in order to make more accurate estimates of milk's bacterial densities (Collado et al., 2009). Knowing total bacterial numbers in milk will be useful to understand bacterial behavior and also, to estimate the bacterial load under infectious situations. This would open new possibilities to develop potential tools to detect problems in the nursing mother. Furthermore, it's known that milk contains a wide range of nutrients, such as lactose, fat or proteins, which can be used as bacterial food source (Petherick, 2010). Milk also contains a variable number of human cells, including epithelial and immune cells, and the number of the latter has been related to lactational mastitis problems (Hassiotou et al., 2013). Thus, the relationship between bacterial load and other factors such as milk developmental stage, nutrient composition, number of somatic immune cells, or bacterial diversity have not been studied in depth.

Therefore, the purpose of the present study was to develop and establish a protocol using molecular techniques and flow cytometry to calculate the exact number of bacteria present in milk at three lactation stages from different mothers, and correlate this bacterial load to the abovementioned factors that could influence it.

## Materials and methods

### Subjects and sampling

A total of 21 healthy Spanish mothers with exclusive breast feeding practices participated in the study and provided samples of breast milk (BM) within 1 month after delivery. Breast-milk samples were collected within 5 days after mothers gave birth (colostrum), between 6 and 15 days (transition) and after 15 days (mature). However, only 57 samples were analyzed, as not all of mothers provided a sample at the three time points. Details of delivery and gestational age were collected after birth. Written informed consent was obtained from the participants and the study protocol was approved by the Ethics Committee of the CSIC (Spanish National Research Council).

Before sample collection, the mothers were given oral and written instructions for standardized collection of samples. Previously, nipples and mammary areola were cleaned with soap and sterile water and soaked in chlorhexidine to reduce bacteria residing on the skin. The milk samples were collected in a sterile tube manually, discarding the first drops, with a sterile milk collection unit. All samples were kept frozen at −20°C until delivery to the laboratory.

### DNA isolation

Milk samples (5–10 mL) were thawed and centrifuged at 4000 × g for 20 min to separate fat and cells from whey. Thereafter, total DNA was isolated from the pellets by using the MasterPure Complete DNA and RNA Purification Kit (Epicenter) according to the manufacturer's instructions with some modifications (Simón-Soro et al., 2015). Two hundred and fifty microliters of saline solution and 250 μl of lysis buffer were added to the pellets, together with Pathogen Lysis Tubes (QIAGEN) glass beads. Both chemical and physical cells disruption was performed after mixing vigorously the samples in a TissueLyser II (QIAGEN) during 5 min at 30 Hz, incubating in dry ice 3 and 5 min at 65°C in a thermoblock, repeating the process 2 times. Fifty microliters of lysozyme (20 mg/ml) and 5 μl of lysostaphin (20 μg/ ml) were added to the tubes, and the samples were incubated for 1 h at 37°C. Two microliters of proteinase K were added and samples were incubated for 15 m at 65°C. The reaction was ended putting tubes on ice, and proteins were precipitated using 350 μl of the protein precipitation agent, discarding the pellets. DNA was precipitated using isopropanol, washed with 70% Ethanol and resuspended with 30 μl TE buffer. The total DNA isolated was quantified with a NanoDrop ND-1000 (ThermoScientific) spectrophotometer.

### Quantitative real-time polymerase chain reaction analysis and bacterial load

qPCR amplification and detection were performed with primers targeted to the fusA gene, a bacterial gene which is present in a single and unique copy per bacterial cell (Santos and Ochman, 2004), making it a more accurate target for bacterial load estimations compared to the 16S rRNA gene, which is present in variable copy numbers among different bacterial species. The use of a single-copy gene in qPCR analysis implies that the number of gene copies equals the number of bacterial cells, improving measures of bacterial densities. In this work, we used modified fusA gene primers from Santos and Ochman (2004), based on multiple alignments with all sequences of this gene in the Ribosomal Database Project Functional Gene Repository (Fish et al., 2013) as available on January 2015, using an annealing temperature of 62°C in a Light Cycler 480 Real-Time PCR System (Roche Technologies). The primer sequences were as follows: 138F- GCTGCAACCATGGACTGGAT, and 293R- TCRATGGTGAAGTCAACGTG. Each reaction mixture of 20 μl was composed of KAPA Sybr Fast qPCR Kit (KAPA Biosistems), 0.4 μl of each primer (10 μM concentration) and 1 μl of template DNA using an annealing temperature of 62°C in a Light Cycler 480 Real-Time PCR System (Roche Technologies). All amplifications were performed in duplicates. The bacterial concentration in each sample was calculated by comparison with the Ct values obtained from standard curves. These were generated using serial 10-fold dilutions of DNA extracted from 10 million bacteria quantified and sorted from a pool of four milk samples from different mothers using a MoFlo XDP cytometer, after mild sonication to separate aggregated cells (Simón-Soro et al., 2015).

### PCR amplification and pyrosequencing

Partial 16S rRNA genes were amplified by PCR with the universal bacterial primers 8F and 785R (Simón-Soro et al., 2014) by the use of high-fidelity AB-Gene DNA polymerase (Thermo Scientific) with an annealing temperature of 52°C and 20 cycles. A secondary amplification was performed by using the purified PCR product as a template, in which the universal primers were modified to contain the pyrosequencing adaptors A and B and an 8-bp “barcode” specific to each sample, following the method used in Benítez-Páez et al. (2013). The final DNA per sample was purified by using an Ultrapure PCR purification kit (Roche), and its concentration was measured by PicoGreen fluorescence in a Modulus 9200 fluorimeter from Turner Biosystems. PCR products were pyrosequenced from the forward primer end using a 454 Life Sciences system, in a GS-FLX sequencer with Titanium chemistry (Roche) at the Foundation for the Promotion of Health and Biomedical Research (FISABIO) in Valencia, Spain. Sequences were deposited in the MG-RAST public repository under the project name “Relationship between milk microbiota, bacterial load, macronutrients, and human cells during lactation” with Accession Numbers 4689674.3-4689703.3.

### Sequence analysis

Sequences with an average quality value <20 and/or with >4 ambiguities in homopolymeric regions in the first 360 flows were excluded from the analysis. Obtained 16S rRNA reads were end-trimmed in 10 pb sliding windows with average quality value >20, then length (200 bp) and quality filtered (average *Q* > 20). Only sequences longer than 400 bp were considered and chimeric reads were eliminated using UCHIME (Edgar et al., 2011). Sequences were assigned to each sample by the 8-bp barcode and phylum-, family-, and genus-level taxonomic assignment of sequences that passed quality control were made using the Ribosomal Database Project classifier software (Wang et al., 2007) within an 80% confidence threshold. Sequences >97% identical were considered to correspond to the same operational taxonomical unit (OTU), representing a group of sequences that presumably correspond to the same species (Yarza et al., 2008). Sequences were clustered at 97% nucleotide identity over 90% sequence alignment length using the CD-hit software (Li and Godzik, 2006). Rarefaction curves were calculated with the RDP pyrosequencing pipeline (Cole et al., 2009) using the same number of randomly selected sequences per group and Chao1 and Shannon estimations (representing species richness and diversity, respectively) were obtained. For those genera found at higher than 1% frequency, a BLASTn (Altschul et al., 1997) was performed against the RDP database, selecting those hits with nucleotide identity values >97% and alignment lengths >400 bp, following (Cabrera-Rubio et al., 2012b).

### Milk composition analysis

We analyzed 38 milk samples from 17 mothers with known bacterial load to elucidate their fat, protein, and lactose composition (% w/w) by spectrophotometry using a MilkoScan FT 6000 (FOSS), and the number of somatic cells (cells/ml) using an Integrated Milk Testing Fossomatic FC(FOSS) cytometer, in LICOVAL, Valencia, Spain.

### Bacterial fractions in milk

Bacterial distribution in human milk was determined after analyzing 10 ml of colostrum (*n* = 9) and mature milk (*n* = 9) samples, using a MoFloXDP cytometer with sorter. Transition samples were not analyzed due to lack of volume availability. Light was produced by an argon laser of 400 nm (blue light) and 200 Mw. First, the machine was calibrated using electromagnetic beads (Fluorospheres, Beckman Coulter Inc.) with known size (1, 3, and 10 μm). Then, events under 3 μm (containing planktonic bacteria) and those over 3 μm (containing human cells) were counted and sorted in two different tubes. DNA was extracted from both fractions for each sample, and qPCR was performed to determine the number of bacteria present in each of them, corresponding to free-living and human cells-associated bacteria. Fluorescence microscopy was performed on a selected number of samples after marking with DAPI dye, and visualized on a Nikon Eclipse E800 microscope. For Scanning Electron Microscopy, samples were kept on Karnovsky solution and further fixed with 1% OsO_4_ in PBS buffer. Samples were then dehydrated with ethanol and critical-point drying, attached to a stub and coated with gold. Images were obtained in a Hitachi S-4800 Scanning electron Microscope with default settings at University of Valencia.

## Results and discussion

### Bacterial load in milk

**Figure 1:**
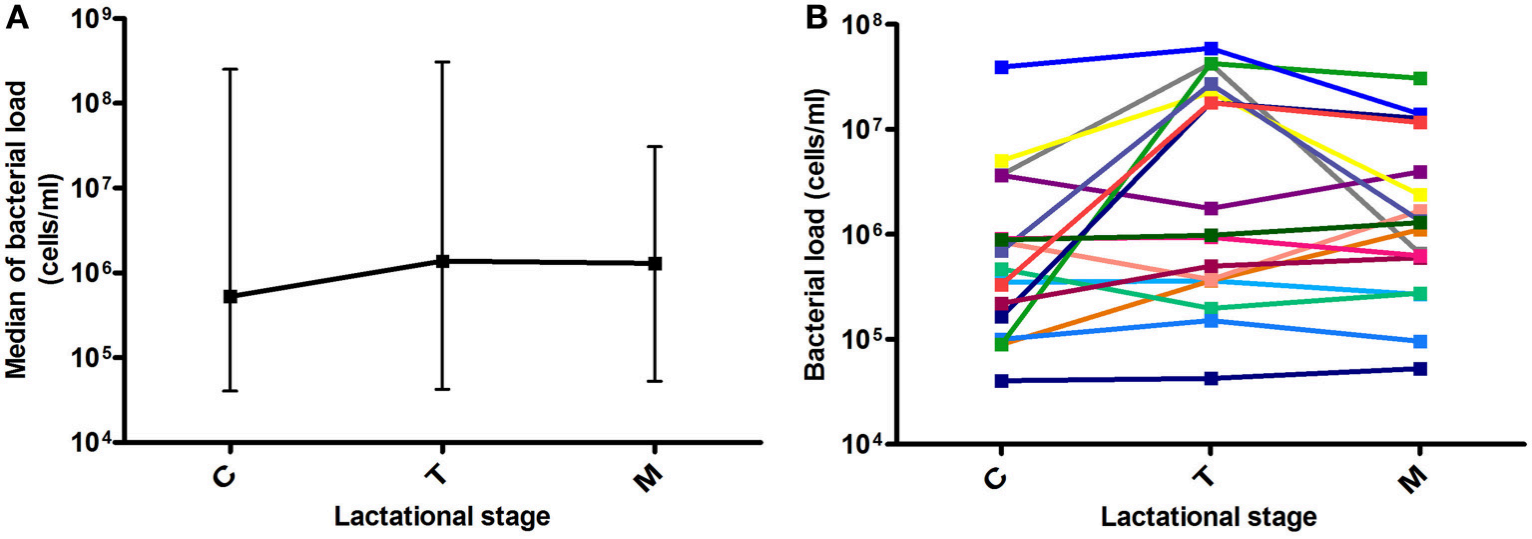
**Bacterial load over lactational stages. (A)** Data show the median with ranges (maximum and minimum values for each group) of bacterial load at the three time points. C, colostrum samples (*n* = 19); T, transition milk samples (*n* = 20); M, mature milk samples (*n* = 17). **(B)** Lines show individual bacterial load for each mother at the three time points (*n* = 17).

Bacterial load values at each milk maturation stage are shown in Figure 1. After analyzing 56 milk samples by qPCR, results showed large individual differences in bacterial load over time between samples from the different mothers and in some cases even within individuals at different time points, indicating that human milk samples are highly variable in microbial content. Median values for colostrum, transition and mature milk were around 10^6^ bacterial cells per ml, with no significant differences between the three time points. Data from other researchers had indicated bacterial densities of 10^3^–10^4^ per ml of breast milk, but they were based on laboratory culture (Heikkilä and Saris, 2003), or on qPCR methods calibrated by culture (Khodayar-Pardo et al., 2014), which account for a limited fraction of total bacteria in human samples. In addition, a significant fraction of microorganisms were found to be adhered to the extracellular matrix of human cells (see Section Bacterial Distribution in Milk Below), which could further prevent the growth on culture media. The molecular approach used in the current manuscript expands these pioneering estimates, allowing now the study of potential relationships between bacterial load and other parameters. Although our molecular-based methods suggest bacterial loads between two and three orders of magnitude higher than those estimated by culture, it has to be taken into account that DNA from non-viable bacteria and extracellular DNA would also be amplified by qPCR, and therefore the real number of viable bacteria would probably be lower.

### Milk bacterial composition across lactation time

**Figure 2:**
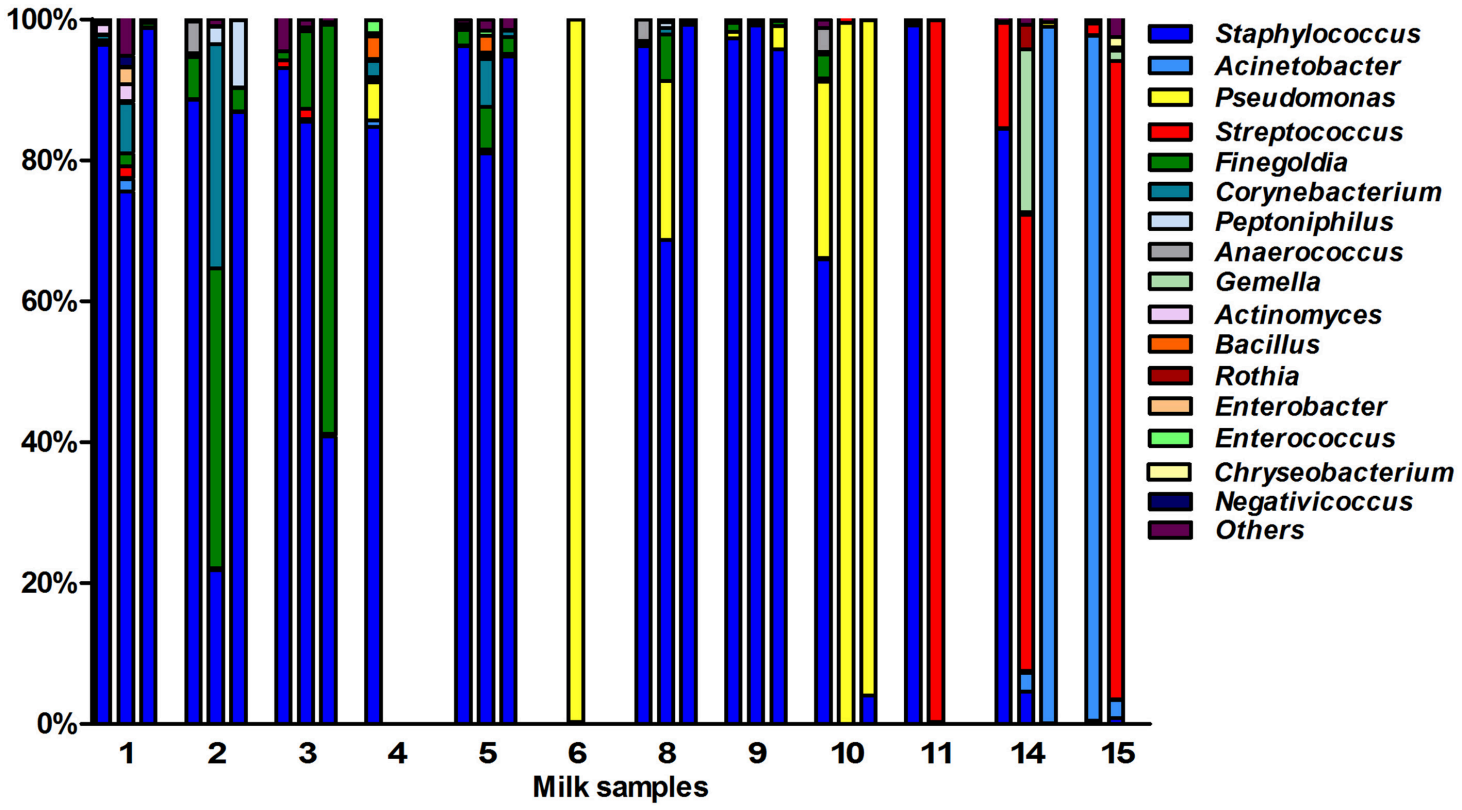
**Bacterial taxonomic composition of human breast milk**. The bars show the proportion of bacterial genera as inferred by PCR amplification and pyrosequencing of the 16S rRNA gene in healthy mothers (*n* = 12). Each number in the X axis represent a donor, with first column representing the colostrum sample, second the transition milk and third the mature milk samples. In some cases, data from the three breastfeeding stages could not be obtained due to sample unavailability or sequencing failure. Bacterial genera that were under 1% were grouped in the “Others” category.

**Figure 3:**
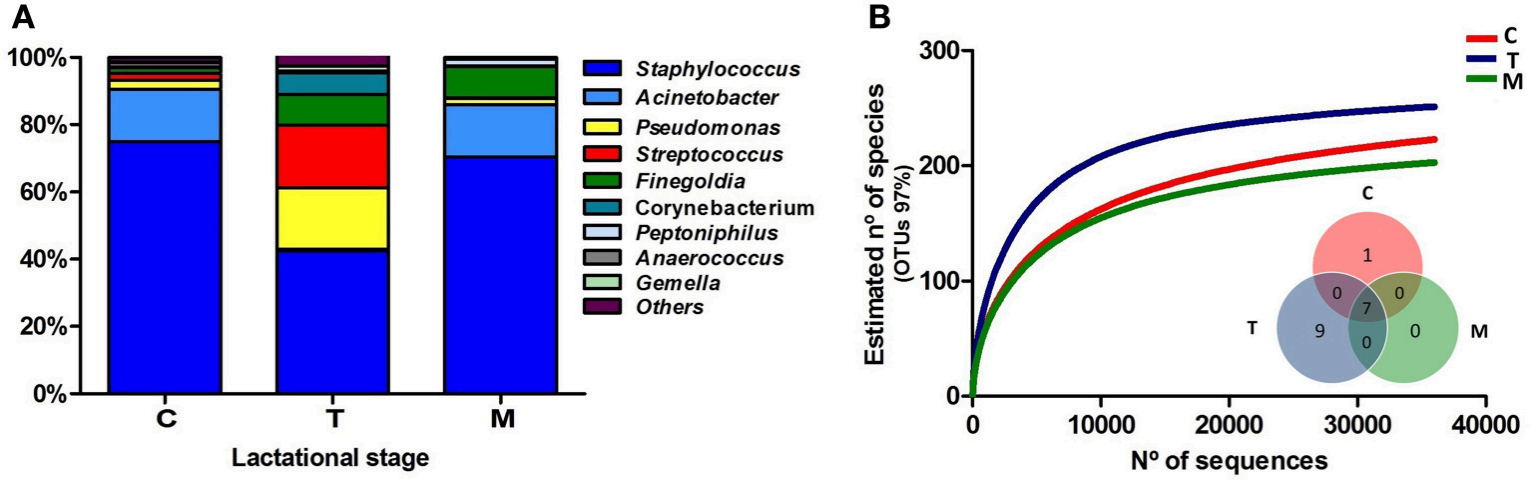
**Bacterial diversity of human breast milk. (A)** Shows the proportion of each bacterial genera in the three lactational-stages, as inferred by PCR amplification and pyrosequencing of the 16S rRNA gene. **(B)** Shows the rarefaction curves of the three groups, relating the sequencing effort with an estimate of the number of bacterial species, as inferred by the number of OTUs. An OTU is a cluster of 16SrRNA sequences that were >95% identical, a conservative estimate for the boundary between species, established at 97% for full-length 16S rRNA sequences. The inlet Venn's diagram shows the number of bacterial genera shared between and unique to the three sample types, excluding bacterial genera present at <1% proportion. Seven genera are shared at the three breastfeeding stages: *Finegoldia, Streptococcus, Corynebacterium, Staphylococcus, Acinetobacter, Peptoniphilus*, and *Pseudomonas*. C, colostrum samples (*n* = 11); T, transition samples (*n* = 11); M, mature samples (*n* = 8).

After quality filtering and length trimming, 174,886 16S rRNA sequences were analyzed, with an average number of taxonomically assigned, high-quality sequences of 4353 reads per sample. The taxonomic assignment of the sequences showed that human breast milk composition is dominated by *Staphylococcaceae*, which account for >62% of the total number of sequences obtained (Figure 2). At the three lactation times, the most common genera was *Staphylococcus*, followed by *Acinetobacter* in colostrum*, Pseudomonas* and *Streptococcus* in transition milk and also *Acinetobacter* in mature milk samples (Figure 3A). Milk from the three lactation points showed different patterns of bacterial diversity, but no statistically significant differences were found between timepoints for any bacterial genus. Rarefaction curves after analyzing 35,000 reads per lactation time point indicated 223 OTUs in colostrum samples, 251 in transition and 203 in mature samples when sequences were clustered at 97% sequence identity (the consensus value for determining species boundaries; Figure 3B). The number of OTUs obtained suggests values of several hundred species in human breast milk, with transition samples having higher diversity than colostrum and mature milk, containing up to nine genera that were only found at that stage (Figure 3B). Similar estimates of several hundred bacterial species were also obtained by other studies (Hunt et al., 2011, Cabrera-Rubio et al., 2012a), confirming that human breast milk is highly diverse. However, most diversity in the samples corresponded to a few bacterial genera, which appeared to be dominant. Among them, we found a core of seven genera that were present at the three time points: *Finegoldia, Streptococcus, Corynebacterium, Staphylococcus, Acinetobacter, Peptoniphilus*, and *Pseudomonas*. Although determining the bacterial species composition with partial 16S rRNA sequences has to be taken with care, the relatively long sequences obtained by pyrosequencing (average read length 718 bp) allowed us to assign reads to the species taxonomic level with some degree of reliability. This analysis revealed that the most common species within Staphylococci was *S. epidermidis*, and *S. aureus* was not detected in these healthy mothers (a full list of species composition can be found in Table 1). It must be underlined that although some bacteria typically associated to human breast milk like *Bifidobacterium* spp (Collado et al., 2009) were detected at low proportions in our samples, this could be due to low amplification efficiency of “universal” primers in these high G+C content taxa (Sim et al., 2012).

**Table 1:** **Prevalence of bacterial genera and species in breast milk samples**.

**Genera**	**Prevalence^a^**	**Species**	**Prevalence^b^**
*Staphylococcus*	24/30	*Staphylococcus epidermidis*	22/24
		*Staphylococcus lugdunensis*	5/24
		*Staphylococcus hominis*	5/24
		*Staphylococcus microti*	3/24
		*Staphylococcus warneri*	1/24
		*Staphylococcus equorum*	1/24
*Streptococcus*	13/30	*Streptococcus mitis*	7/13
		*Streptococcus infantis*	6/13
		*Streptococcus cristatus*	5/13
		*Streptococcus salivarius*	4/13
		*Streptococcus mutans*	3/13
		*Streptococcus sanguinis*	3/13
		*Streptococcus gordonii*	1/13
		*Streptococcus sanguinosus*	1/13
*Finegoldia*	9/30	*Finegoldia magna*	9/9
*Pseudomonas*	8/30	*Pseudomonas deceptionensis*	3/7
		*Pseudomonas fragi*	3/7
		*Pseudomonas meridiana*	3/7
		*Pseudomonas gessardii*	2/7
		*Pseudomonas moorei*	1/7
		*Pseudomonas japonica*	1/7
		*Pseudomonas sasplenii*	1/7
*Acinetobacter*	7/30	*Acinetobacter haemolyticus*	4/7
		*Acinetobacter junii*	2/7
		*Acinetobacter ursingii*	2/7
		*Acinetobacter lwoffii*	2/7
		*Acinetobacter parvus*	1/7
		*Acinetobacter guillouiae*	1/7
		*Acinetobacter pittii*	1/7
		*Pseudomonas alcaliphila*	1/7
*Anaerococcus*	5/30	*Anaerococcus octavius*	5/5
		*Anaerococcus murdochii*	1/5
		*Anaerococcus prevotii*	1/5
*Actinomyces*	4/30	*Actinomyces radingae*	3/4
		*Actinomyces neuii*	2/4
*Enterobacter*	4/30	*Enterobacter cancerogenus*	2/3
		*Enterobacter aerogenes*	1/3
		*Enterobacter hormaechei*	1/3
		*Enterobacter asburiae*	1/3
		*Enterobacter kobei*	1/3
*Peptoniphilus*	3/30	*Peptoniphilus lacrimalis*	1/3
		*Peptoniphilus gorbachii*	1/3
		*Peptoniphilus harei*	1/3
*Gemella*	3/30	*Gemella haemolysans*	3/3
*Rothia*	3/30	*Rothia mucilaginosa*	3/3
*Corynebacterium*	2/30	*Corynebacterium simulans*	1/2
		*Corynebacterium xerosis*	1/2
		*Corynebacterium amycolatum*	1/2
*Bacillus*	2/30	*Bacillus thuringiensis*	1/2
		*Bacillus circulans*	1/2
		*Bacillus megaterium*	1/2
*Chryseobacterium*	1/30	*Chryseobacterium daeguense*	1/1

*Assignment to the species taxonomic level was performed by BLASTn selecting only alignments >300 bp and sequence identity >97%*.

a *Data indicate the number of samples containing the indicated genus*.

b *Data indicate the number of samples containing the indicated species referred to the number of samples containing the corresponding genus*.

It is interesting to note that the bacterial genera found in our samples, which were obtained from Spanish mothers, was different to those found in other high-throughput sequencing studies from American or Finnish milk samples (Hunt et al., 2011, Cabrera-Rubio et al., 2012a), suggesting that geographic, genetic, and dietary factors could be influencing microbial diversity in breast milk.

### Relationship between bacterial load and milk's composition and diversity

**Figure 4:**
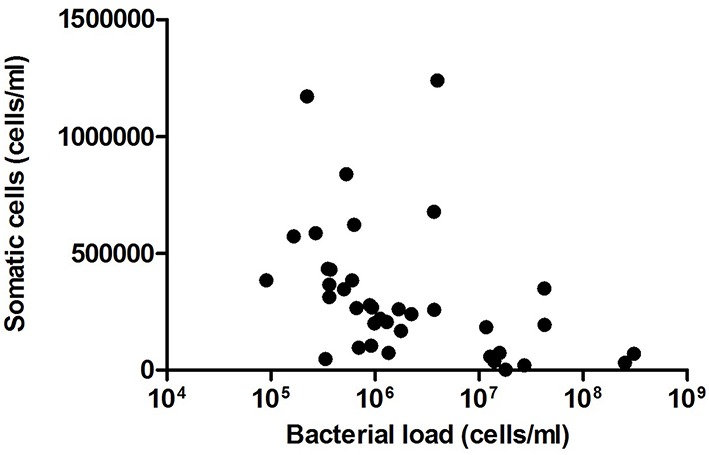
**Relationship between number of somatic cells and bacterial load in milk samples**. The graph shows a comparison between the number of bacterial cells per milliliter (estimated by qPCR) and the number of somatic cells per milliliter, estimated with an Integrated Milk Testing Fossomatic 5000 (FOSS) cytometer. (*n* = 38, *R*^2^ = 0.0066). C, colostrum samples (*n* = 12); T, transition samples (*n* = 15); M, mature samples (*n* = 11).

**Figure 5:**
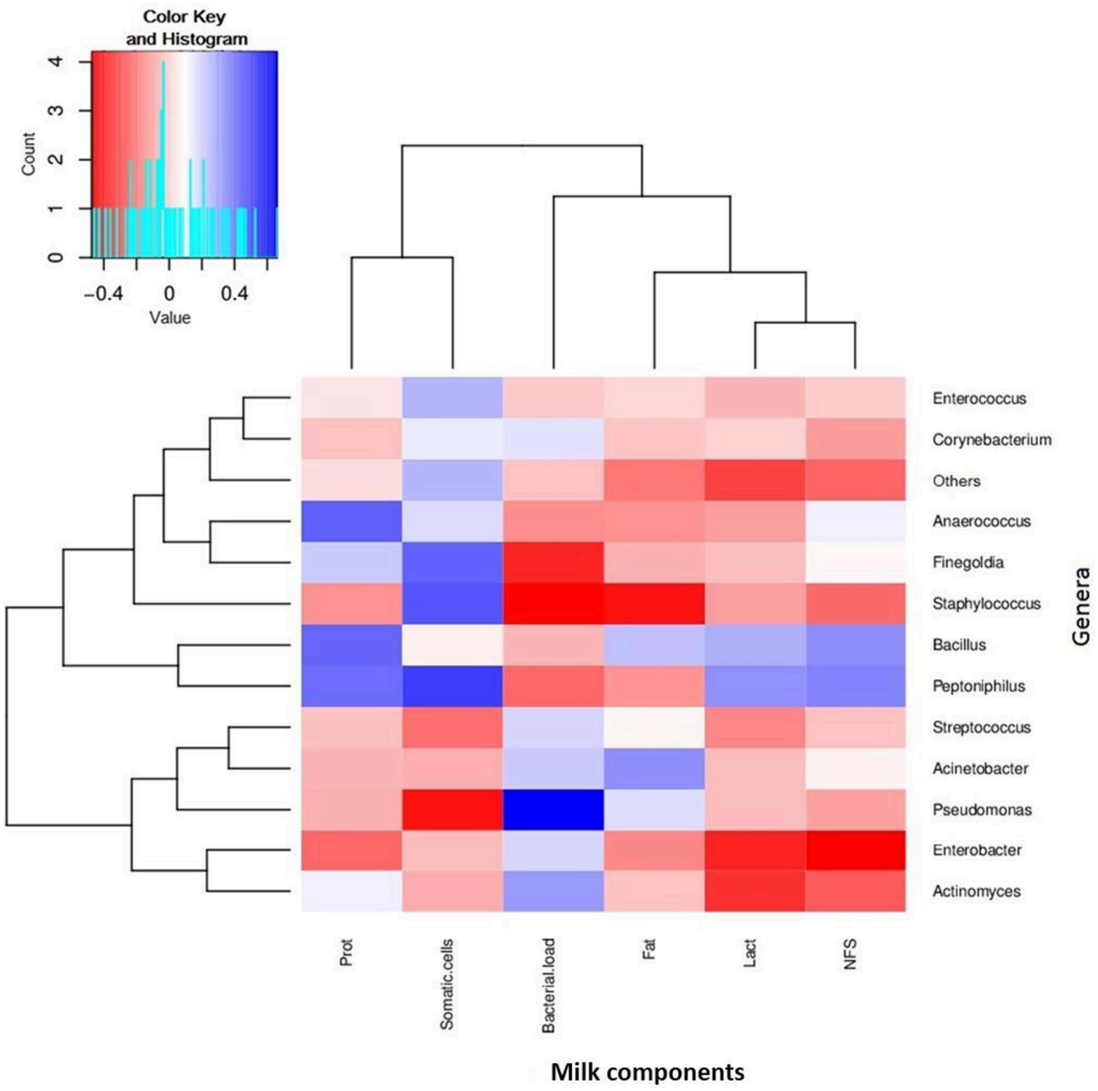
**Relationships between bacterial composition and nutritional or cellular content of human breastmilk**. The figure shows a heatmap where samples have been clustered according to its compositional profile. Bacterial genera appear color-coded according to their under- (red) or over-representation (blue) in the samples, and its proportion is correlated to the amount of protein content (indicated as “prot” in the figure), fat content (Fat), lactose content (Lact), and non-fatty solid content (NFS), as well as the density of bacterial and human somatic cells. *n* = 30.

After comparing the number of somatic cells and bacterial load in the same samples, no significant correlation was found (Figure 4). Given that the number of somatic cells in milk is considered the gold standard for detecting infection (e.g., lactational mastitis) in farm animals (Olechnowicz and Jaśkowski, 2012), the absence of a somatic cell increase in our samples suggests a lack of significant immune response. Thus, the data presented in the current work suggest that high counts of bacteria in milk are not associated with infection in these healthy mothers without lactation problems. However, a positive correlation was found between the proportion of the common mastitis pathogen *Staphylococcus* and the number of somatic cells (Pearson correlation coefficient: 0.48, *p* = 0.0457). Given that a negative relationship was found between the proportion of *Staphylococcus* and the total bacterial load (correlation coefficient: −0.456, *p* = 0.056), the data suggest that it is not the number of bacteria but the specific composition of the milk microbiota that could be inducing an immune response in the mammary gland, although the major mastitis pathogen *S. aureus* was not detected in our samples (Table 1). Other bacteria appeared to show a positive relationship with the number of somatic cells were *Peptoniphilus* and *Finegoldia* (Figure 5), although the correlations were not statistically significant in these cases. It has to be kept in mind that breast milk contains several anti-inflammatory (He et al., 2016) that could partly reduce somatic cells counts.

**Figure 6:**
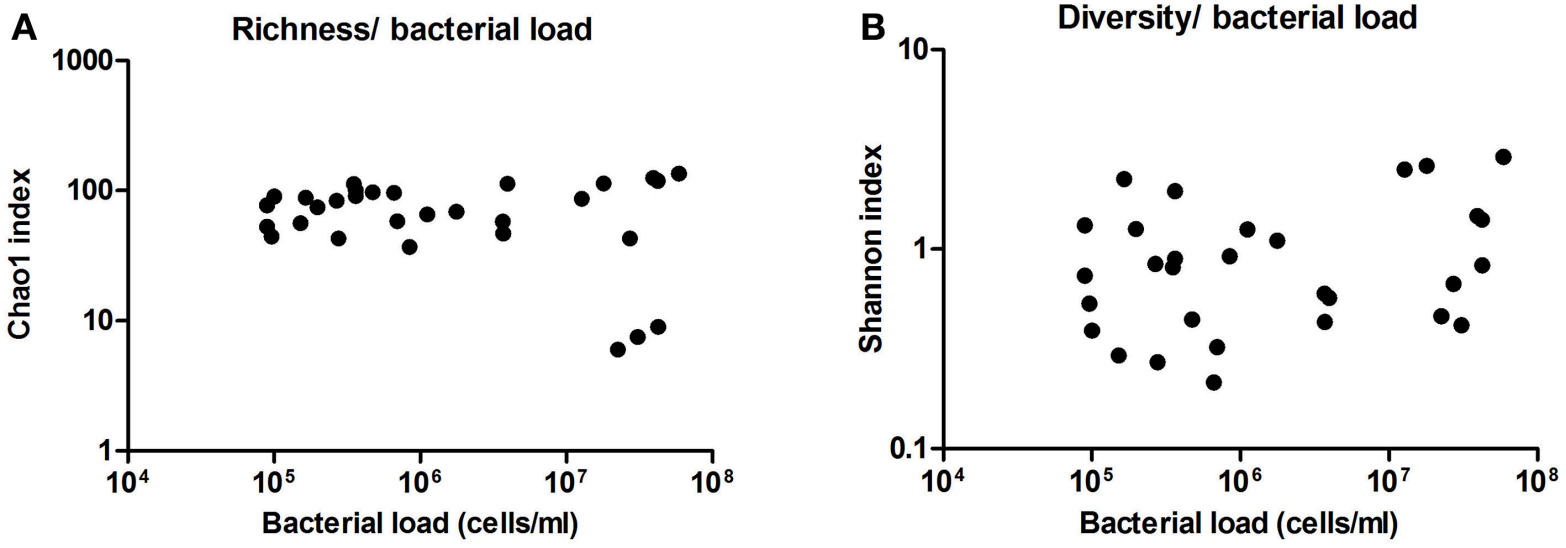
**Richness and diversity of milk samples. (A)** Shows the richness in the samples as inferred by computation of Chao1 index, compared with bacterial load in cells per ml, as estimated by qPCR. **(B)** Shows the diversity in the samples as inferred by Shannon index, compared with bacterial load. (*n* = 30 in both cases).

Additionally, we analyzed the diversity and richness of the bacteria present in these samples by the statistic indexes “Shannon” and “Chao1,” respectively. Neither diversity nor richness increased or decreased significantly with bacterial load (Figure 6). This also supports a lack of subclinical or sub-acute mastitis, as an increase of a few dominant bacteria would be expected in case of infection, and suggests that milk microbiota is not activating an immune response in the host, although inflammatory markers have not been measured.

We also analyzed fat, protein, lactose and non-fatty solid fractions in milk, and compared them with the bacterial load, in order to find any possible correlations (Supplementary Figure 1). No significant correlations were found with the number of bacteria per ml. However, some positive and negative relationships were found between some nutrients and specific bacterial genera (Figure 5). For instance, the amount of proteins were positively correlated with the proportion of *Bacillus, Peptoniphilus*, and *Anaerococcus* in the samples, whereas lactose levels were negatively correlated with *Enterobacter* and *Actinomyces*, indicating potential prebiotic and antagonistic effects for bacterial growth, which should be evaluated in bigger sample sizes. In the case of fat, whose content in milk is known to increase through breastfeeding, it was negatively correlated with the proportion of *Staphylococcus* (Pearson correlation coefficient: −0.425, *p* = 0.0443), and therefore if this negative relationship is confirmed in larger cohorts, high fat content in milk could potentially be protective of mastitis risk.

### Bacterial distribution in milk

**Figure 7:**
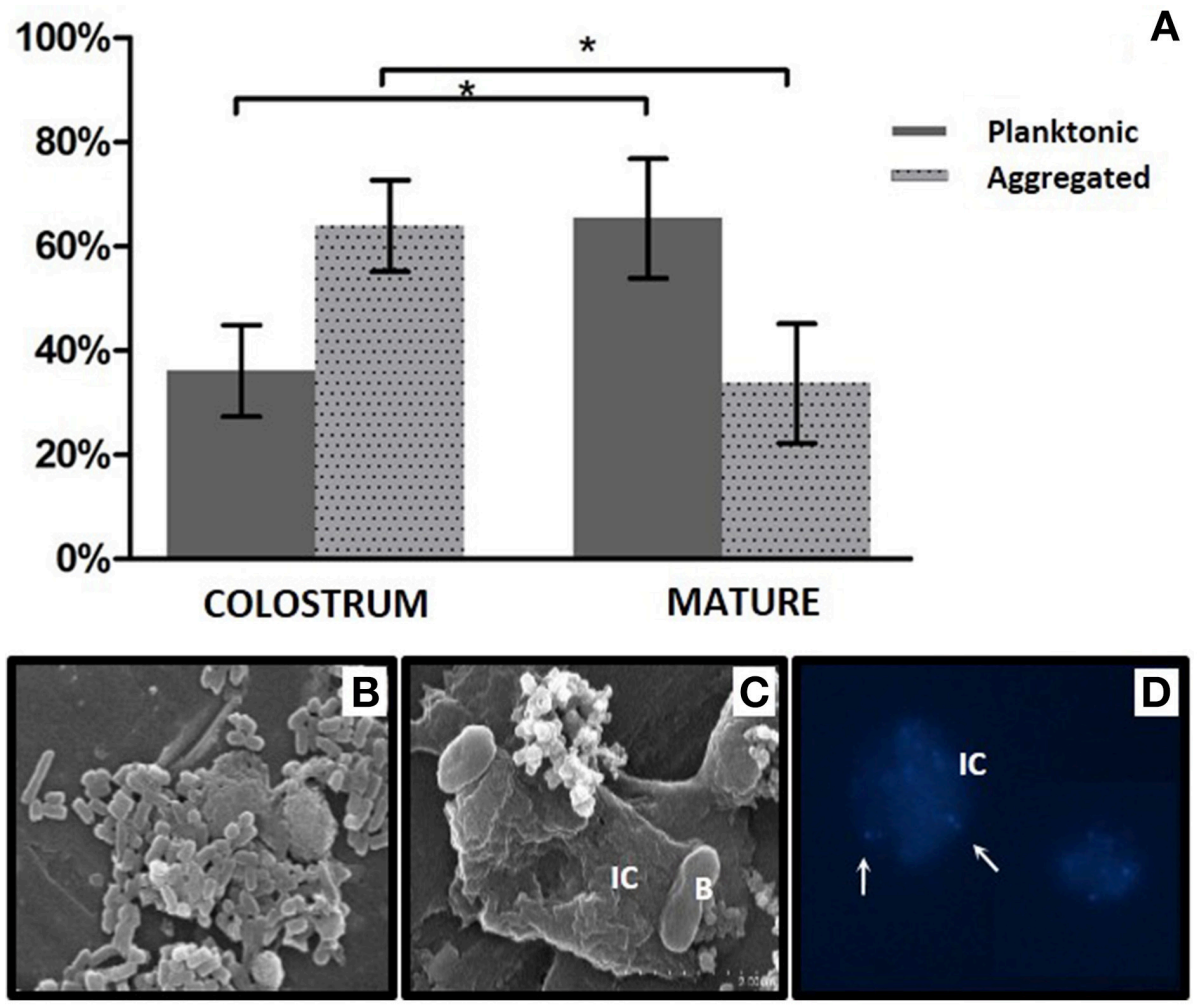
**Bacterial fractions in human breast milk. (A)** Proportion of bacteria present in a free-living, “planktonic” state and aggregated to human immune cells in colostrum and mature milk samples. Bacteria from 10 ml of milk were counted and sorted by size and complexity using a Moflo cytometer. *indicates a *p* < 0.05, Mann–Whitney test. **(B)** Planktonic bacteria in milk observed by SEM microscopy. **(C)** Bacteria associated to human immune cells, observed with SEM microscopy. **(D)** Bacteria associated to human immune cells, observed with fluorescence microscopy. DNA was stained with DAPI fluorophore. Bacteria are indicated with arrows. IC, human immune cell; B, bacteria.

Bacterial loads in planktonic and human cell-associated fractions of nine samples of colostrum and nine samples of mature milk were calculated, showing that the microorganisms were present in both fractions, although aggregated bacteria appeared to be more abundant in colostrum (65.75%), and planktonic bacteria were found to be more abundant in mature samples (63.92%; Figure 7A). Mann-Whitney statistical tests showed significant differences (*p* < 0.05) between the two time points (but not within the same time point) for both free and human cell-associated bacteria. The high proportion of bacteria associated with human immune cells was confirmed by fluorescence and Scanning Electron microscopy (Figures 7B–D). Bacteria in the aggregated fraction seemed to be adhered to the membrane of human cells (identified as immune cells according to their shape and size) but not intracellular. We confirmed the presence of live bacteria moving inside the extracellular matrix of immune cells (Supplementary Video). Bacterial cells in this extracellular matrix have also been observed in blood samples from pregnant mothers by other researchers (Donnet-Hughes et al., 2010). An “entero-mammary pathway” has been proposed to explain the translocation of bacteria to the mammary gland through blood and/or lymph stream through its association to human immune cells (Martín et al., 2004). If this translocation process is confirmed, the milk cell-bacterial association described here could be a consequence of such a relationship. An alternative explanation would be that bacteria originated from skin and the oral cavity of the lactating child invades the mammary gland and binds to immune cells without eliciting a response (Hagi et al., 2013). Future studies should determine the kind of immune cells involved in the observed bacterial adhesion and the nature of the bacteria-human recognition (Langa, 2006; Perez et al., 2007), including the identification of which microorganisms are free and which ones are human cell-associated.

## Conclusion

Our estimates of bacterial load provided by molecular methods indicate that a lactating infant feeding 800 ml of breastmilk per day could ingest 10^7^–10^8^ bacterial cells daily, about 100 times higher than previous estimates based on laboratory culture methodologies. Our data show that samples with higher bacterial load in healthy mothers do not suffer from lower diversity, as it would be expected from microbial infections. In addition, no correlation between human and bacterial cells was found in milk, suggesting that milk microbiota is not seen as an infection by the mother's immune system, and that the immune response is directed toward specific microorganisms such as *Staphylococcus*. Furthermore, specific relationships between macronutrients and specific bacteria have been described. However, more studies with higher number of samples are needed to confirm and identify key interactions between bacteria and nutrients and their potential impact in infant health. Thus, the biological function of these potentially symbiotic bacteria for infant health could be relevant, including a role in the development of their immune system, and should be elucidated.

## Author contributions

AM and MC conceived the study project. AB, MC, and AM designed experiments. AB performed experiments and analyzed the data. All authors contributed to interpretation, drafted, and critically revised the manuscript. All authors gave final approval and agree to be accountable for all aspects of the work.

### Conflict of interest statement

The authors declare that the research was conducted in the absence of any commercial or financial relationships that could be construed as a potential conflict of interest.

## Supplementary material

Supplementary Video **Time-lapse photography of a human milk immune cell containing a live bacterial cell embedded in its surface**. Apart from being free-living, between 40 and 60% of milk bacteria were found attached to human cells, as estimated by qPCR of plancktonic and aggregated bacteria.

Supplementary Figure 1 **Relationship between bacterial load and macronutrients in human milk**. The graphs show the comparison between bacterial load and: **(A)** protein, **(B)** lactose, **(C)** fat, and **(D)** non fatty solids in the samples (*n* = 38).
