# Supplementary figures and images for: Relationship between Milk Microbiota, Bacterial Load, Macronutrients, and Human Cells during Lactation
Source: Front Microbiol. 2016 Apr 20;7:492. doi: 10.3389/fmicb.2016.00492 (PMC4837678; doi:10.3389/fmicb.2016.00492)

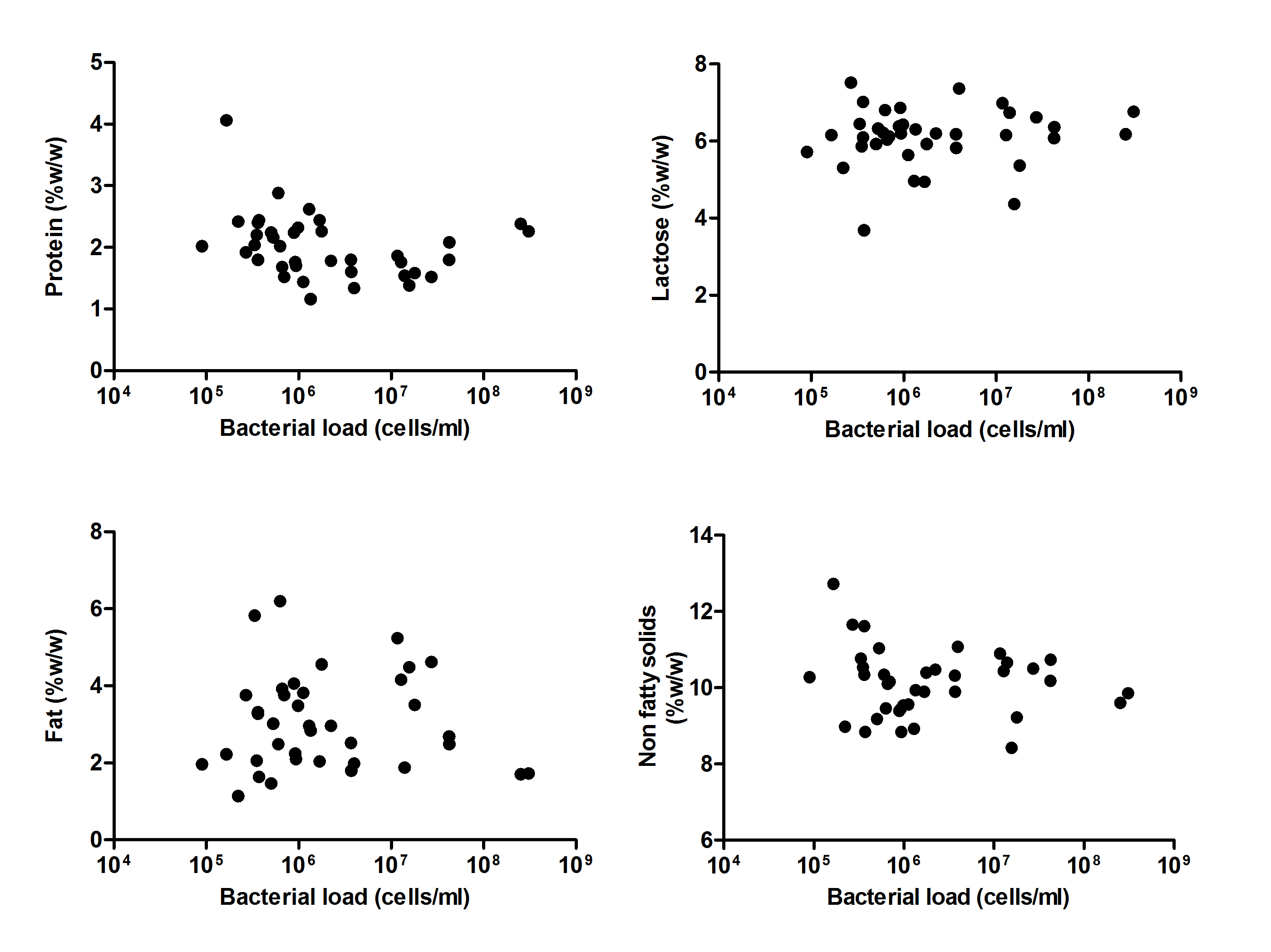

Supplement: Supplementary Figure 1 — Relationship between bacterial load and macronutrients in human milk. The graphs show the comparison between bacterial load and: (A) protein, (B) lactose, (C) fat, and (D) non fatty solids in the samples (n = 38). [file Image1.JPEG]
